# Evaluation of the Quality Characteristics and Development of a Puffed-Rice Snack Enriched with Honeybee (*Apis mellifera* L.) Drone Pupae Powder

**DOI:** 10.3390/foods11111599

**Published:** 2022-05-28

**Authors:** Woo-Hee Cho, Jung-Min Park, Eun-Ji Kim, Md. Mohibbullah, Jae-Suk Choi

**Affiliations:** 1 Seafood Research Center, IACF, Silla University, 606, Advanced Seafood Processing Complex, Wonyang-ro, Amnam-dong, Seo-gu, Busan 49277, Korea; ftrnd3@silla.ac.kr (W.-H.C.); mmohib.fpht@sau.edu.bd (M.M.); 2 With Food Corp., 204, Busan Inno-Biz Center, Mandeok 3-ro 16beon-gil, Buk-gu, Busan 46570, Korea; pjm_kdessert@naver.com (J.-M.P.); 1abmiracle@naver.com (E.-J.K.); 3 Department of Fishing and Post Harvest Technology, Sher-e-Bangla Agricultural University, Sher-e-Bangla Nagar, Dhaka 1207, Bangladesh; 4 Department of Food Biotechnology, Silla University, 140, Baegyang-daero 700beon-gil, Sasang-gu, Busan 46958, Korea

**Keywords:** puffed-rice snack, drone pupae, quality characteristics, *Apis mellifera* L., nutritional profile

## Abstract

Edible insect ingredients have gained importance as environmental-friendly energy sources world-wide; the honeybee (*Apis mellifera* L.) drone pupae has gained prominence as a nutritional material. In this study, bee drone pupae were processed under different heating and drying conditions and incorporated into a puffed-rice snack with honey. The sensory, physicochemical, nutritional and microbial qualities of drone pupae powders were tested. The deep-fried and hot-air dried powder was selected; the values of 5.54% (powder) and 2.13% (honey) were obtained on optimization with honey by response surface methodology. Subsequently, the puffed-rice snack product enriched with drone pupae powder was stored at different temperatures for 180 days. The prepared product showed a higher content of proteins, fats, amino acids, and fatty acids compared to the control. The high content of a few minerals were maintained in the processed powder and the product, whereas heavy metals were not detected. The storage test indicated acceptable sensory qualities and safety results, considering important quality parameters. Thus, drone pupae powder and the developed product can be consumed as nutritional food materials; the quality characteristics can be improved through optimal processing.

## 1. Introduction

Honeybee (*Apis mellifera* L.) drone pupae are rich sources of protein and other essential nutrients (such as carbohydrates, fats, amino acids, minerals, and vitamins) and can be a valuable food ingredient [1]. Bee drone pupae are used as nutritious and high-protein food in Korea, Japan, China, the United States, and many European countries, and can be added to various dishes (such as, soups, confectioneries, and baked goods) [2]. To popularize drone pupae powder as a food material, the RDA (Rural Development Administration) of South Korea has selected 11 drone pupae powder-added food items (including cookies, sesame tapioca bread, chocolates, and shakes) and published their recipe book [3]. Furthermore, a jelly-type energy supplement prepared using drone pupae powder is already in existence, while several studies confirm the application of ground insects in familiar edible items (such as, bread, biscuits, and so on) [4,5]. Additionally, it is used as a raw material for chocolate, confectionery, alcoholic beverages, and functional foods [2,6,7].

The Food and Agriculture Organization (FAO) has published reports on the nutritional value, collection, storage, quality control, and use of bee larvae, pupae, and adult insects in food, medicine, and cosmetics (Krell, 1996) [8]. Honeybee (*Apis mellifera* L.) drone pupae were registered as a food ingredient in Korea by the MFDS (Ministry of Food Drug Safety) in July 2020 [9].

To promote the human consumption of honeybee drone pupae, in addition to the development of novel promising functional foods and pharmaceuticals, the direct application of insect powder in food is under consideration. Various snack products enriched with insects, such as, grasshoppers (*Sphenarium purpurascens* Ch.) [10], migratory locusts (*Locusta migratoria*) [11], lesser mealworms (*Alphitobius diaperinus*) [12] and yellow mealworms (*Tenebrio molitor*) have been developed recently [13]. These insects, being rich in protein, increase the protein content of grain-based snacks. There are no detailed reports on grain-based snacks enriched with honeybee (*Apis mellifera* L.) drone pupae.

Grain-based snacks incorporating insects mainly use wheat [13], maize (*Zea mays* L.) [10], and chickpea [11]. Wheat, a protein-rich resource, is widely used in the food industry because of its low price, convenience of procurement, easy processibility, etc. However, gluten-free alternatives are required for people with wheat-gluten allergies [14]. Rice is rapidly gaining popularity as an alternative to wheat; soft, low-allergenic, and easily digestible puffed-rice snacks are particularly popular [15]. 

In this study, the best drone pupae processing method was identified, a puffed-rice snack enriched with honeybee (*Apis mellifera* L.) drone pupae powder was developed, and its quality characteristics were evaluated after a storage test.

## 2. Materials and Methods

### 2.1. Materials

Honeybee (*Apis mellifera* L.) drone pupae (20 days old) were purchased from the Cho-Won beekeeping farm (Pohang, Gyeongsangbuk-do, Korea). The honeycomb containing the collected bee drone pupae was rapidly frozen using a deep-freezer (DF3503S, ilShinBioBase Co., Ltd., Dongducheon, Gyeonggi-do, Korea) and stored at −20 °C until experimentation. Puffed rice (Woori food Co., Anseong, Gyeonggi-do, Korea) was used for the development of the snack product (prepared at about 250 °C). Soybean oil, white sugar, and starch syrup were purchased from the CJ Cheiljedang Corporation (Seoul, Korea). Organic solvents and indicators of the highest grade were used for the physicochemical analysis (Sigma-Aldrich Co. LLC., St. Louis, MO, USA).

### 2.2. Drone Pupae Preparation and Pre-Treatment for Processing to Powder

**Figure 1:**
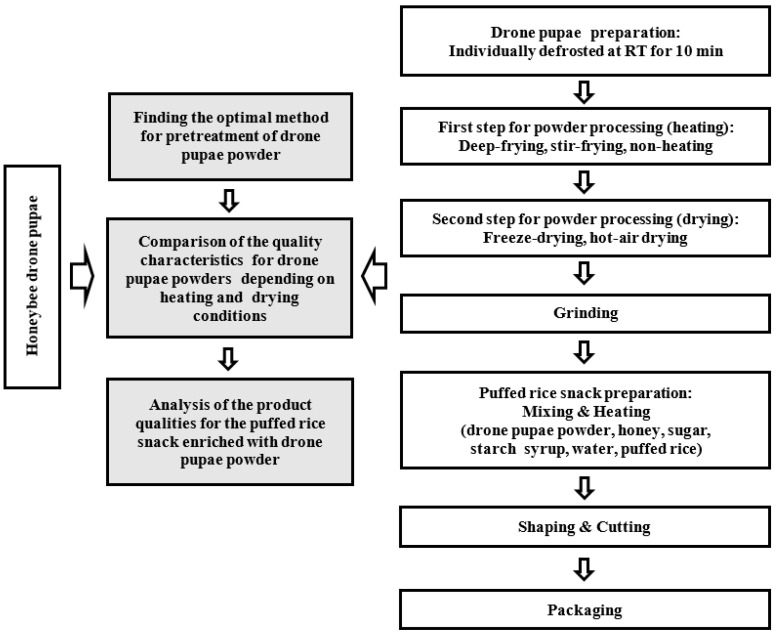
Schematic design of the drone pupae powder and the developed product research (RT; room temperature; 15–20 °C).

The processing flow shown in Figure 1 was used to process drone pupae powder and develop the rice snack. The frozen drone pupae were individually picked up from the hive and thawed at room temperature (RT) within 10 min, immediately after being taken out of the freezer. After the removal of water with a paper towel (Kimtech Science Wipers, Yuhan-Kimberly, Seoul, Korea), the thawed drone pupae were used in the next process.

Heating-drying was used for processing to powder form. Three groups of samples underwent deep-frying with oil (150 °C, 12 min), stir-frying without oil (180 °C, 3 min), or non-heating. The heated sample groups were treated by hot-air drying and freeze-drying. A hot-air drying machine (P-IOV864, Labmate, Gumi, Gyeongsangbuk-do, Korea) was used at 70 °C for 14 h, while a freeze-dryer (Lyoph-prido 20R, ilShinBioBase Co., Ltd., Dongducheon, Gyeonggi-do, Korea) was used for two days until the sample dried up to about 4.5% (*w*/*w*) of its moisture content.

The dried drone pupae were pulverized in a grinder (HMF-3100S, Hanil Electric Co., Ltd., Seoul, Korea) for about 10 min and then filtered using a 7 mm sieve. The prepared drone pupae powder was sealed in a zipper pack and stored in a freezer (DF3503S, ilShinBioBase Co., Ltd. Dongducheon, Gyeonggi-do, Korea) at −35 °C or less until further experimentation.

### 2.3. Development of the Puffed-Rice Snack Enriched with Drone Pupae Powder

To prepare the syrup base, 50 g of starch syrup, sugar, and water were mixed together in a pot and boiled over low heat until the weight of the syrup was reduced to 100 g. After turning off the heat, puffed rice (50 g) was added and stirred with the syrup base to ensure smooth mixing. Subsequently, drone pupae powder and honey were mixed into the puffed-rice mixture in different concentrations according to the central composite design (CCD) and response surface methodology (RSM). Finally, the puffed-rice mixture was placed in a rectangular stainless-steel tray, flattened by a rolling stick to a 2.5 cm-thick sheet, and cooled (at RT) for 10 min. The puffed-rice snack enriched with drone pupae powder was cut into 3.5 cm (H) × 3.5 cm (W) × 2.5 cm (T) pieces and stored in a zipper bag at RT.

### 2.4. Optimization of the Mixing Conditions for Drone Pupae Powder and Honey with Puffed Rice

Based on the descriptive sensory test, the mixing conditions (%, *w*/*v*) for drone pupae powder and honey with puffed rice were optimized using the response surface methodology (RSM). Table 1 shows the mixing conditions of the prepared materials in terms of the central composition using different variables and code values (−1.414, −1, 0, +1, +1.414). The concentrations of drone pupae powder (X_1_) and honey (X_2_) with a base rice snack were set as independent variables (factors), while the descriptive sensory terms for nutty aroma (Y_1_) and sweetness taste (Y_2_) were applied as dependent variables (responses) using a 10-point strong level scale. With this experimental design, a comparison using the descriptive sensory analysis was tested with a series of snack samples. MINITAB 18 (Minitab Inc., State College, PA, USA) was used for the model calculation of each response, using the following Equation (1):

(1) $$Y=\beta_{0}+\beta_{1}X_{1}+\beta_{2}X_{2}+\beta_{3}X_{1}^{2}+\beta_{4}X_{2}^{2}+\beta_{5}X_{1}X_{2}$$

The formula contains the coefficients for regression β_0_ (intercept), β_1,2_ (linear), β_3,4_ (quadratic), and β_5_ (interaction). The final model was reduced in terms of significance (R^2^) by eliminating the insignificant terms. To optimize with these models, Minitab ver. 19.0 software (Minitab Inc, State College, PA, USA) was used for an analysis of variance (ANOVA) and experimental validation of the computed conditions.

### 2.5. Yield

The yield of the drone pupae powder was calculated by dividing the weight of the dried powder by that of the control drone pupae (frozen; 700 g). Weights were recorded on an electronic scale (WH-1A, Wessglobal, Co., Ltd., Seoul, Korea) after drying the pretreated drone pupae powders in an oven (VS-1202D3, Vision Scientific Co., Ltd., Bucheon, Korea).

### 2.6. Color

The method of Kang et al. [16] was used to measure the color values of the drone pupae powders, using a color difference meter (CM-700d Spectrophotometer; Konica Minolta Sensing Inc. Tokyo, Japan). Hunter system values, including L (lightness), a (redness), and b (yellowness) were monitored using SpectraMagic software (version 2.11; Minolta Cyber Chrome Inc., Osaka, Japan). The overall color difference (Δ*E*) was made between treatment groups of drone pupae and calculated using the following formula: Δ*E* = ((ΔL)^2^ + (Δa)^2^ + (Δb)^2^)^1/2^.

### 2.7. Odor Intensity

The odor intensity was measured according to the method of Kang et al. [17]. Each prepared drone pupae powder (5 g) was placed in a 50 mL conical tube (SPL Life Science Co., Ltd., Pocheon, Gyeonggi-do, Korea) with a control group (the frozen drone pupae). The suction port of the odor-concentration meter (XP-329R, New Cosmos Electric Co., Ltd., Osaka, Japan) was inserted into the conical tube and sealed with parafilm to enable odor retention. Subsequently, the odor intensity of each drone pupae powder was measured; the measurement mode of the odor concentration meter was set to batch, with a unit of odor intensity (VCI; Volatile Component Intensity).

### 2.8. pH Value

A pH meter (ST3100, Ohaus, Parsippany, NJ, USA) was used to measure the pH after adding 25 mL of distilled water to 5 g of the sample and mixing. Each sample was independently measured three times and expressed as an average pH value.

### 2.9. Acid Value

The acid value was analyzed according to the Food Code [18]. Extracted lipid (5 g) from the sample was taken in an Erlenmeyer flask (1000 mL), followed by the addition of 100 mL of ethanol and ether-mixed solution (1:2, *v*/*v*). After adding 1% phenolphthalein solution as indicator, the solution was titrated using 0.1 N ethanolic potassium hydroxide until a pale red color persisted for 30 s.

### 2.10. Volatile Basic Nitrogen (VBN)

The volatile basic nitrogen (VBN) content was measured by the micro-diffusion method using the Conway unit mentioned in the Food Code (MFDS, 2021) [18].

### 2.11. Thiobarbituric Acid Reactive Substance (TBARS)

Thiobarbituric acid reactive substance (TBARS) values were measured by the method of Buege and Aust [19]. After adding 12.5 mL of 20% trichloroacetic acid (TCA) in 2 M phosphoric acid to 5 g of each sample, and homogenization at 1000 rpm for 1 min using a homogenizer (SHG-15D, SciLab Co., Ltd., Seoul, Korea), the volumes were adjusted to 25 mL each using distilled water. The homogenates were centrifuged at 1600× *g* for 15 min at 4 °C using a centrifuge (Labogene 416, Gyrozen Co., Ltd., Gimpo-si, Korea). Subsequently, the supernatants were filtered with filter paper (70 mm, Toyo Roshi Kaisha Ltd., Tokyo, Japan); 2 mL of each filtrate was mixed with 0.005 M thiobarbituric acid (TBA) solution, heated to 95 °C for 30 min in a water bath (JSWB-22TL, JS Research Inc., Gongju-si, Korea), and cooled to room temperature. The quantification of TBARS involved measuring absorbance at 530 nm using a microplate reader (SPECTRO star-nano, BMG Labtech, Ortenberg, Germany).

### 2.12. Sensory Evaluation

The sensory evaluation was performed for different purposes by two different methods: the optimizing process (descriptive analysis) and controlling quality (affective analysis). To establish optimal conditions, eight trained panelists (from Woori Corp, Pyongtaek, Korea), equipped to provide detailed information about bee-processing products participated in the descriptive tests. Each descriptive term (darkness color, brownness color, nutty aroma, sweetness taste, burnt taste, bitter taste, sticky texture, rough texture) was evaluated by a 10-point strong level scale according to the glossary and standard scores shown in Table 2. After the storage test, a 9-point hedonic scale (1: I hate it very much, 9: I like it very much) evaluation (appearance, odor, taste, texture, overall acceptance) was carried out by 21 semi-trained panelists belonging to the Silla University Industry-Academic Cooperation Foundation.

The former method is in accordance with a standard descriptive analysis in the International Organization for Standardization (ISO 13299: 2016) [20], while the latter follows the guidelines of the shelf-life test in the MFDS. These sensory tests have been approved by the Institutional Review Board of Silla University (IRB approval number; 1041449-202108-HR-002).

### 2.13. Total Amino-Acid Content

The total amino-acid content was measured according to a method (protocol 994.12) of the AOAC (Association of Official Analytical Chemists) [21]. One gram of the sample was placed in a test tube and mixed with 15 mL of 6 N HCl solution. Subsequently, the test sample was sealed under reduced pressure and acid hydrolyzed at 110 °C for 24 h in a drying oven (VS-1202D3, Vision Scientific Co., Ltd., Bucheon, Korea). The decomposed solution was filtered with a glass filter, and the filtrate was concentrated under reduced pressure at 55 °C to completely evaporate HCl and water using a rotary vacuum evaporator (EYELA N-1000, Riakikai Co., Ltd., Tokyo, Japan). The concentrated sample was aliquoted with a sodium citrate buffer (pH 2.2) and filtered by a 0.45 µm-thick membrane filter. The total amino-acid content was measured using an automatic amino acid analyzer (Biochrom 20, Pharmacia Biotech Ltd., Cambridge, UK).

### 2.14. Fatty-Acid Composition

The fatty-acid composition was analyzed by the following protocol. First, lipids were extracted from the samples by the method of Bligh and Dyer [22]. Subsequently, after methyl esterification of fatty acids according to a method of the AOCS (American Oil Chemists’ Society) [23,24], the fatty-acid composition was analyzed using a gas chromatography instrument (Shimadzu GC-2010; carrier gas, He; detector, FID) equipped with a capillary column (Supelcowax-10 fused silica wall-coated open tubular column, 30 m × 0.25 mm Id, Supelco Japan Ltd. Tokyo, Japan). The injector and detector (FID) temperatures were set to 250 °C, respectively, while the column temperature was increased to 230 °C and maintained for 15 min. He (1.0 kg/cm^2^) was used as the carrier gas with a 1:50 split ratio. The fatty-acid composition was determined by comparing the recorded retention times with those of standard fatty acids (Applied Science Lab. Co., Baldwin Park, CA, USA).

### 2.15. Analysis of Nutrition and Minerals

According to the standard test methods (protocol 925.09, 923.03, 979.09, 962.09, and 923.05) of AOAC [25], 14 nutrients (calories, sodium, carbohydrate, sugar, dietary fiber, crude fat, trans fat, saturated fat, cholesterol, crude protein, vitamin D, potassium, iron, and calcium) of the puffed-rice snack enriched with drone pupae powder were analyzed. The minerals (Na, Ca, K, Fe, P, Mg, Zn, and Cu) of the selected drone pupae powder were analyzed using a method of the Food Code (MFDS, 2021) using inductively coupled plasma (ICP; Optima 5300 DV; Perkin Elmer, Waltham, MA, USA) [18].

### 2.16. Heavy Metals

Four heavy metals (lead, cadmium, mercury, and arsenic) were analyzed according to a method of the Food Code [26]. Each sample (10 g) was heated in a microwave oven (MARS 6, CEM Corporation, Matthews, NC, USA) at 450 °C until completely carbonized, and then homogenized using a homogenizer (SHG-15D, SciLab Co., Ltd., Seoul, Korea). Subsequently, they were dissolved in 5 N nitric acid solutions to obtain at least 20 mL of experimental solution for each sample. The concentrations (mg/kg) of lead, cadmium, and arsenic were measured by inductively coupled plasma (ICP; Optima 5300 DV; Perkin Elmer, Waltham, MA, USA) in triplicates, whereas 1 g of the sample was analyzed using a mercury analyzer (MA-2; Nippon Instruments Corporation, Tokyo, Japan) to determine the mercury concentration.

### 2.17. Microbial Analysis

The total bacteria count was measured using methods (protocol 990.12 and 991.14) of the AOAC (2002) [27]. The sample (10 g) was collected in a sterile pack (3M Co., Ltd., Saint Paul, MN, USA), mixed with 900 mL of 0.85% NaCl sterilized saline, and inoculated on a 3M dry film after ten-times dilution. The number of colonies were counted after culturing at 35 ± 1 °C for 24–48 h; the valid number of colonies (showing a red dot with gas) were counted to detect *E. coli* and the total coliform group.

### 2.18. Moisture Content

The moisture content of the samples were measured using a method of the Food Code [18]. Each sample (5 g) was dried at 105 °C for 24 h using a drying oven, cooled in a desiccator for 30 min, and weighed. The moisture content (*w*/*w* % of wet basis) was calculated using the following equation: Moisture (%) = ((g of initial weight)—(g of final weight))/(g of initial weight) × 100.

### 2.19. Statistical Analysis

All experiments (except the optimization by response surface methodology) were performed thrice and the measured values were statistically analyzed by one-way ANOVA along with *t*-tests using the SPSS version 23.0 (IBM Corp., Armonk, NY, USA). The significant difference was evaluated at *p*-values < 0.05.

## 3. Results and Discussion

### 3.1. Establishment of Optimal Processing Conditions for Drone Pupae Powder

To develop the puffed-rice snack enriched with drone pupae powder, the heating and drying conditions were first tested for processing from drone pupae to powder. Through a comparison of the quality characteristics of the treated drone pupae powders, the optimal processing conditions for their pre-treatment were determined. Here, different heating methods (stir-frying without oil, deep-frying with oil, and unheated condition) were used, followed by drying (hot-air drying, freeze-drying), for three groups of drone pupae powder. Finally, six groups of heated and dried drone pupae were pulverized, and the different quality characteristics (sensory characteristics, yield, color, odor intensity, acid value, pH, TBARS, and VBN) were evaluated.

#### 3.1.1. Sensory Evaluation Results of Drone Pupae Powders Using the Descriptive Test

Table 3 shows the results of the descriptive analysis for color, aroma, taste, and texture of the drone pupae powders. The color parameter was evaluated considering darkness and brownness. The highest darkness levels were scored by the DFD (8.00) and DHD (7.38) groups, with an insignificant difference (*p* < 0.05); statistically, the DHD (8.88) and DFD (8.13) groups exhibited the highest brownness values, while the UFD (1.50) and SFD (2.38) groups exhibited the lowest values. Both DHD (7.38) and DFD (8.00) exhibited the strongest points for nutty-aroma, with an insignificant difference. On heating protein compounds in food to the temperature range of 130–140 °C, different colors and flavors are obtained by the Maillard reaction, which cause browning and impart a nutty smell to food (Lin et al., 2009) [28].

In this study, the sensory panelists also scored on the burnt and bitter taste to account for any changes in surface and chemical conditions due to the heating treatments. The UHD (2.63), UFD (2.13) and SFD (2.38) groups showed significantly lower burnt tastes than the other three powders (*p* < 0.05). The highest bitterness (taste) was observed in the UFD (4.38) and SFD (4.25) groups, which were significantly higher than the other powders. Each powder, treated by a different combination of frying (deep frying, stir-frying, control) and drying (freeze-drying, hot-air drying) conditions, exhibited different texture characteristics (sticky, rough). The DHD (6.25) and DFD (5.13) groups showed the highest values for sticky texture. Thus, cooking oil (used for deep-frying) considerably influenced sticky texture, while the rough texture was also attributed to deep-frying (*p* < 0.05).

#### 3.1.2. Yields of Drone Pupae Powders

The yield values for the drone pupae, considering the heating and drying method, are shown in Table 4. The post pre-treatment weight and corresponding yields for all the powders were: DFD (222 g; 31.71%), DHD (206 g; 29.43%), UFD (172 g; 24.57%), SFD (167 g; 23.86%), UHD (156 g; 22.29%), and SHD (150 g; 21.43%). Considering the statistical differences, DFD (31.71%) and UHD (22.29%) exhibited the highest and lowest yields, respectively (*p* < 0.05). Overall, the deep-fried samples exhibited higher yields than the other groups; this could be attributed to the presence of some frying oil portion and its degraded polar compounds absorbed by food during deep-frying [29].

#### 3.1.3. Instrumental Sensory Characteristic Results of Drone Pupae Powders

**Figure 2:**
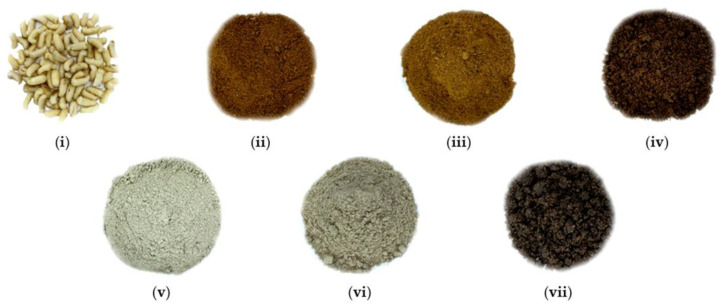
Sample groups of drone pupae (*Apis mellifera* L.) powder processed under different conditions: (**i**) Control drone pupae; (**ii**) UHD; (**iii**) SHD; (**iv**) DHD; (**v**) UFD; (**vi**) SFD; (**vii**) DFD.

The color values (L, a, b, and Δ*E*) and odor-intensity values of the instrumental sensory characteristics of variously treated drone pupae powders are shown in Table 5, while the actual sample pictures of the powders are shown in Figure 2. All the measured color values showed significant differences with each other (*p* < 0.05). After the control drone pupae (56.84), the UFD group exhibited the highest L value (41.28). Additionally, the Δ*E* (21.87) for UFD was closest to that of the control (32.92), indicating high similarity with the original color. It has been reported in a previous study (Hwang and Kim, 2020) [30] that a similarly treated (unheated and freeze-dried) silkworm powder shows a higher L value than other differently treated powder samples. The a-values (indicating red color) for the freeze-dried drone pupae powders (UFD, 2.26; SFD, 2.96; DFD, 5.82) were lower than those of the hot-air dried powders (UHD, 8.83; SHD, 7.16; DHD, 8.21), regardless of the heating conditions (unheated, stir-frying, deep-frying).

The results were consistent with the colors observed in actual photographs; UHD, SHD, and DHD exhibited some red color while UFD, SFD and DFD appeared mainly achromatic (white, grey, and black). The appearance of non-achromatic colors (such as brown or red) with high a-values could be attributed to melanoidin, chemically derived by the Maillard reaction. Similar to the trend of a-value distribution, the b-values (indicating yellowness) of the hot-air dried samples (UHD, 19.37; SHD, 17.63; DHD, 12.03) were higher than those of the freeze-dried ones (UFD, 12.02; DFD, 11.41), except for SFD (12.88).

The treated drone pupae powders exhibited odor intensities in the range of 300–400 VCI, except UFD (453.67). The unheated powders (UHD, UFD) showed the highest odor-intensity values (382.33 and 453.67, respectively) in their groups. Thus, the additional heat treatments of stir-frying and deep-frying could have a higher effect on decreasing odor intensity levels than non-heating.

#### 3.1.4. Physicochemical Quality Characteristics of Drone Pupae Powders

**Figure 3:**
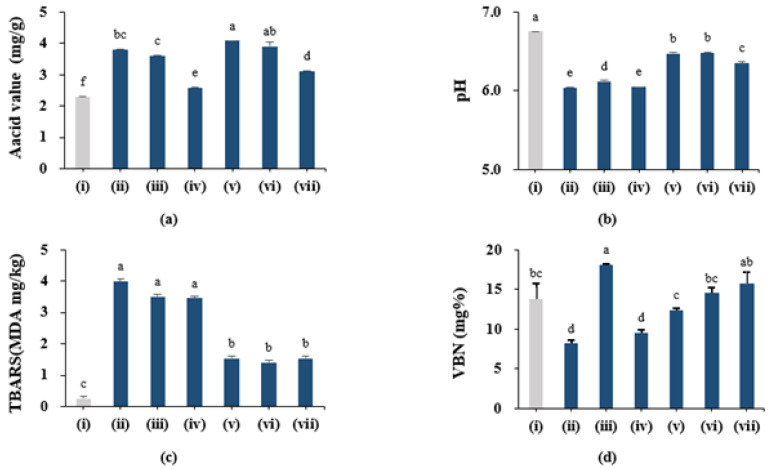
The chemical quality parameters ((**a**), acid value; (**b**), pH; (**c**), TBARS; (**d**), VBN) of the drone pupae powders depending on the pretreatment conditions. (i) The control drone pupae; (ii) UHD; (iii) SHD; (iv) DHD; (v) UFD; (vi) SFD; (vii) DFD. Values are mean ± SD. Different letters (a–f) in each column indicate significant differences among the means by the Tukey’s test (*p* < 0.05).

Figure 3 shows the physicochemical quality characteristics (acid value, pH, VBN, TBARS) of the drone pupae powders and the control (freeze-thawed raw drone pupae), depending on the combination of the three heating conditions and two drying methods used for processing.

As shown in Figure 3a, the acid values of UHD (3.79 mg/g), SHD (3.59 mg/g), DHD (2.57 mg/g), UFD (4.08 mg/g), SFD (3.9 mg/g), and DFD (3.1 mg/g) were higher than that of the control drone pupae (2.29 mg/g). Herein, the control (freeze-thawed raw drone pupae) was measured using the raw drone pupae (2.92 mg/g) according to a previously published study [31]. The high content of unsaturated fatty acids in the drone pupae indicated that the heating and drying processes affected lipid oxidation. However, the acid values of the six drone pupae powders were within the acceptable limit (5.0 mg/g) for processed insect foods in the Food Code [18], indicating their applicability as food material. Furthermore, samples that underwent less oil treatment exhibited higher acid values, regardless of the drying method. However, no studies on drone pupae processed by different frying methods have been reported so far. Thus, as reported in a study by Andrikopoulos et al. [32] on potato processing, pan-frying was assumed to increase the acid value to a greater extent than deep-frying.

The hydrogen-ion concentrations (pH values) for the six drone pupae powders are shown in Figure 3b; all the powders exhibited lower values (6.04–6.48) than that of the control drone pupae (6.75). Overall, the average pH value of the hot air-dried powders (UHD, SHD, DHD; 6.07) were lower than that of the freeze-dried powders (UFD, SFD, DFD; 6.43), and the difference between the two groups was statistically significant (*p* < 0.05). Here, this could be due to a difference in the drying temperature of the two groups; hot-air drying increased the acidity levels to a greater extent.

The TBARS value is an indicator of the degree of lipid oxidation, showing the intensity of malonaldehyde (MDA) produced by the oxidation of fat and thio-barbituric acid [33]. As shown in Figure 3c, the levels of MDA for UHD (3.99), SHD (3.50), and DHD (3.45) were higher than those for UFD (1.54), SFD (1.41), and DFD (1.55), respectively, with significant differences (*p* < 0.05). Thus, the freeze-drying treatment exhibited better stability, as indicated by lipid oxidation, than the hot-air drying treatment, in the processing of drone pupae powder. In the present study, the moisture levels of the hot-air dried powders were remarkably decreased due to their long drying time (14 h) at 70 °C, which could increase the MDA levels and make the lipids unstable [34]. Al-Kahtani et al. [35] have reported that the acceptable limit of TBARS for meat products is 3.0 MDA mg/kg, and lipids do not undergo significant oxidation under this level. Although three hot-air dried drone pupae powders (UHD, SHD, DHD) showed higher TBARS values than the suggested limit, they did not exhibit a bitter taste or unnatural odor due to lipid oxidation, as reported by the sensory panelists, indicating edible-food quality.

Volatile basic nitrogen (VBN) is a general term for volatile amines (such as, ammonia, nitrogen, and trimethylamine) and is an indicator of the decomposition quality of proteins [36]. As the drone pupae has a high protein content, the level of decomposition could be an indicator of its protein-content quality. Figure 3d shows the VBN values of six drone pupae powders with the control. The highest and lowest VBN values were measured in SHD (18.08 mg/100 g) and UHD (8.17 mg%), respectively. Additionally, due to the high value for SHD, the variation of VBN did not exhibit a regular pattern. Fresh meat exhibits VBN values in the range of 5–10 mg%, while a value higher than 30 mg% indicates spoilage, according to Jin et al. [37]. Notably, these recommendations are applicable for general food types, and a different specific standard should be used while considering insect-based foods. No standard for insect-based foods and materials has been suggested to date; however, the VBN values of the drone pupae powders were within the general-food acceptable limits.

In this study, the acid and VBN values were used to determine the optimal heating and drying conditions for processing drone pupae to powder, as they indicate lipid oxidation and protein-content quality. DHD showed the lowest acid value (2.57 mg/g) and VBN level (9.57 mg%). Therefore, the optimal heating and drying conditions for processing drone pupae were selected to be deep-frying and hot-air drying (DHD), and further experiments were conducted using the DHD powder.

### 3.2. Analysis of Microbial Quality Characteristics and Moisture Content of the Optimal Drone Pupae Powder

Table 6 summarizes the total bacteria count (TBC), *E.* *coli*, total coliform group (TCG), and moisture content of the optimal drone pupae powder (DHD) and the control drone pupae. For both samples, the level of TBC did not exceed the recommended value (5 log CFU/g) suggested in the Food Code, while *E. coli* and TCG were not detected. The moisture content of the control material was 51.9%, which was about 11 times higher than that of the optimal drone pupae powder (4.54%) (*p* < 0.05). Kim et al. [38] have reported that the freeze-dried powder of drone pupae (16–20 days old) shows 8.79% moisture content; this is higher than the value obtained for DHD here. This could be attributed to the higher effectiveness of the freeze-drying method compared to hot-air drying; furthermore, deep-frying increases the evaporation rate. The moisture content of the final product (the puffed-rice snack enriched with drone pupae powder) and control snack were 10% and 6.3%, respectively, as indicated by further experimentation.

### 3.3. The Nutritional Composition Results of the Optimal Drone Pupae Powder

#### 3.3.1. Amino Acids

The amino-acid composition of the optimal drone pupae powder (DHD) is summarized in Table 7, along with the recommended daily requirement of essential amino acids according to the World Health Organization (WHO) and the MOHW (Korea). Considering essential amino acids (EAAs), with 15.65 g/100 g of the total EAA, aromatic amino acids (AAAs; tyrosine and phenylalanine) (3.08 g/100 g) were mainly present, followed by leucine (2.89 g/100 g), lysine (2.19 g/100 g), and valine (2.14 g/100 g). The total non-essential amino acids (NAAs) accounted for 18.07 g/100 g (a value that is higher than the sum of the EAAs); glutamic acid (5.43 g/100 g) and aspartate (3.40 g/100 g) were predominantly present.

Thus, the EAA value for the optimal drone pupae powder accounted for 67.5% and 101.6% of the recommended daily requirement according to WHO and MOHW, respectively. The percentages of BCAA (branched-chain amino acid), such as, isoleucine, leucine, and valine, were significantly higher while considering the recommendations of the MOHW than those of WHO. Overall, the optimal drone pupae powder exhibited a lower amino-acid content than freeze-dried drone pupae powders reported in previous studies [39,40], while it exhibited similar values as an oven-dried sample reported in a previous study [41].

#### 3.3.2. Fatty Acids

Table 8 summarizes the fatty-acid composition of the optimal drone pupae powder (DHD) according to a percentage of total fatty acids. Among the saturated fatty acids (32.65%, *w*/*w*), palmitic acid (24.22%, *w*/*w*) occurred predominantly, while oleic acid (32.19% *w*/*w*) and linoleic acid (31.14%, *w*/*w*) mainly accounted for the total unsaturated fatty acid (UFA) content of 67.34% (*w*/*w*). These results are different from those reported in previous studies [39,41], wherein SFA exhibits a higher percentage than UFA. In this study, the tested drone pupae powder was prepared using deep-frying, and could be affected by the high linoleic acid in soybean oil [42]. According to the NNR (Nordic Nutrition Recommendations, 2012) [43], the UFA percentage is recommended to be above two thirds of the total fatty-acid content; the optimal drone pupae powder used here met this criterion.

#### 3.3.3. Minerals and Heavy Metals

Table 9 summarizes the results of mineral and heavy-metal analysis for the optimal drone pupae powder (DHD) according to the recommendations of the MFDS and international standards. The drone pupae powder showed the following values for the eight tested minerals: sodium (41.90 mg), calcium (0.03 g), potassium (1.04 g), iron (5 mg), phosphorus (0.57 g), magnesium (0.07 g), zinc (4.44 mg), and copper (1.19 mg). According to Kim et al. [44], these minerals are not obtained by themselves and are stored in honey and pollen.

In this study, four minerals (sodium, calcium, potassium, iron) were evaluated by standard nutrition facts while general mineral compounds were used to estimate the other four (phosphorus, magnesium, zinc, copper). Here, phosphorus exhibited the highest percentage (0.57 g; 81.4%) according to the recommended amount (>0.7 g) by the MFDS and international standards. The amounts of heavy metals were within the acceptable limits according to the MFDS and international recommendations: lead (0.01 mg), cadmium (0.01 mg), mercury (0.01 mg or less), and arsenic (0.01 mg).

### 3.4. Processing Optimization of the Puffed-Rice Snack Product Using Optimal Drone Pupae Powder and Honey by RSM

**Figure 4:**
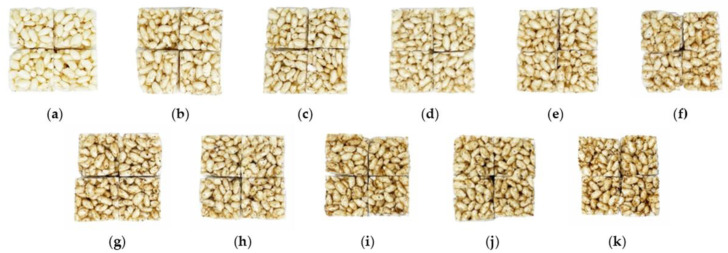
The puffed-rice snacks with different concentrations (*w*/*w*%) of drone pupae powder: (**a**) Control; (**b**) 1%; (**c**) 2%; (**d**) 3%; (**e**) 4%; (**f**) 5%; (**g**) 6%; (**h**) 7%; (**i**) 8%; (**j**) 9%; (**k**) 10%.

For the central composite design (CCD) of response surface methodology, the expected concentration (zero point in Table 1) of the optimal drone pupae powder was selected as the result of the preliminary experiment in the middle of study; this confirmed the acceptable color, smell, and taste depending on the mixed concentrations (1, 2, 3, 4, 5, 6, 7, 8, 9, and 10%) of drone pupae powder with the product, as shown in Figure 4. Subsequently, 5% of drone pupae powder was determined as the zero point for CCD, followed by optimization.

Table 10 summarizes the experimental results for the responses Y_1_ and Y_2_ for the optimization of the puffed-rice snack prepared by processing with different concentrations of drone pupae powder (X_1_) and honey (X_2_). According to these responses, the regression formulae for the response surface model were computed as shown:

Y_1_ (nutty aroma) = 7.963 − 0.487 X_1_ − 0639 X_2_ − 0.169 X_1_ X_1_ − 0.200 X_2_ X_2_

Y_2_ (sweetness taste) = 8.003 − 0.3018 X_1_ + 0.5711 X_2_ + 0.001 X_1_ X_1_ − 0.89 X_2_ X_2_

The statistical correlations for both the models (Y_1_ and Y_2_) were satisfied with the standard *p*-values (<0.05), and they showed high R^2^ values, as shown in Table 11. Lack of fits for the respective models were insignificant (showing *p*-values higher than 0.05), indicating that the models were organized using suitable correlations. Therefore, the exported models could be used to derive optimal conditions for the targeted response qualities (which are written in bold in Table 1).

**Figure 5:**
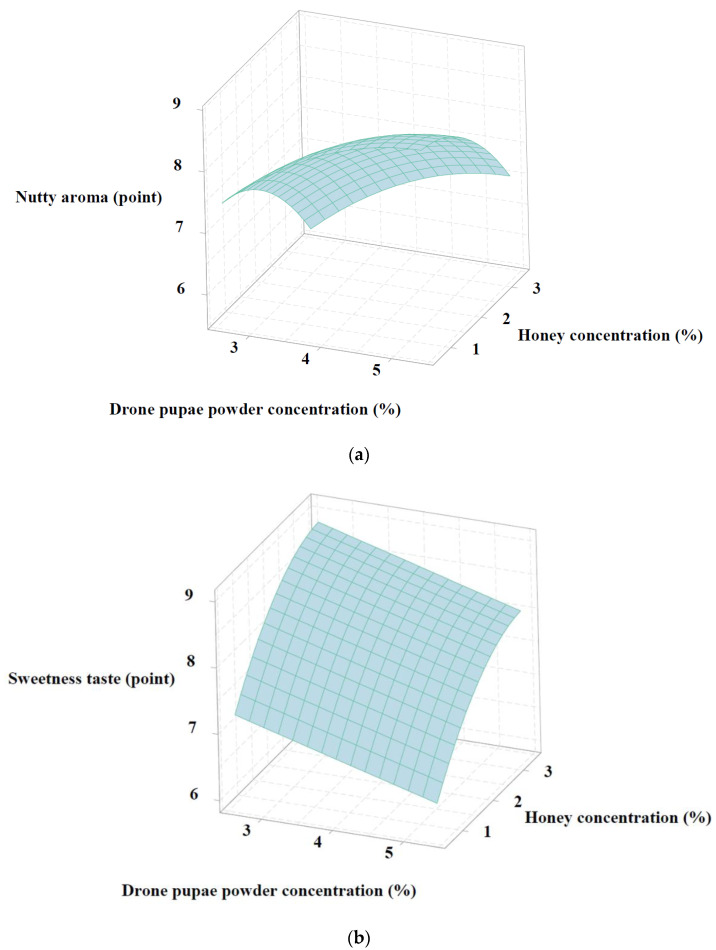
Three-dimensional response surface plots of the puffed-rice snack with respect to drone pupae powder and honey concentrations: (**a**) Nutty aroma; (**b**) Sweetness taste.

As shown in Figure 5a,b, the nutty aroma (Y1) increased on increasing the percentage of drone pupae powder (X_1_), while mixing a higher concentration of honey (X_2_) increased the sweetness taste (Y_2_). Normally, honey used for food processing has a high effect on sweetness due to its high level of fructose (about 75–80%), which is a strong sweetener for human beings (Aparna and Rajalakshmi, 2009) [45]. Furthermore, in this study, adding high concentrations of insect powder (such as drone pupae) in food increased the nutty scent, as the preparatory processes using heat caused the Maillard reaction (Jensen et al., 2016) [46]. The sensory panelists reported a strong nutty aroma and sweet flavor in the puffed-rice snack with a high percentage of drone pupae powder and honey.

Table 12 summarizes the optimal mixing condition for drone pupae powder and honey with the puffed-rice snack, with the expected response values from references, along with the validation results. According to Table 2, the targeted nutty aroma was the level of parched cereal powder (8 point), while chocolate (8 point) was set as the goal score for sweetness taste. Based on model optimization, the predicted responses were 7.992 (nutty aroma) and 7.997 (sweetness taste) on using 5.54% (drone pupae powder) and 2.13% (honey), showing good desirability values. In accordance with the RSM process, these conditions were practically validated with additional experimentation, whereby nutty aroma and sweetness taste were rated at 8.19 and 8.43, respectively (almost equivalent to the predicted values). Thus, this condition was determined as the final mixing concentration and was applied in the subsequent steps.

### 3.5. Product Quality Characteristics of the Puffed-Rice Snack Enriched with Drone Pupae Powder

#### 3.5.1. Nutrition

Table 13 shows the contents of 14 nutrients in the control and drone-pupae-powder enriched puffed-rice snack product as percentages of daily intake recommendations according to the FDA (USA) and MFDS (Korea). The protein (4.45 g) and fat (4.60 g) content of the puffed-rice snack enriched with drone pupae powder was higher than those of the control (2.36 g and 1.06 g, respectively). Thus, the addition of drone pupae powder to food increased its protein and fat content; this is in agreement with a previous study by Biró et al. [47] in which the insect-enriched snack shows increasing protein and fat values with increasing insect concentration. Furthermore, the developed snack product exhibited a high cholesterol value (17.16 mg) due to its high fat content. Considering the suggested daily intake recommendations, the developed product possessed high percentages (76.4% and 38.2%) of sugar content (considering the limits according to the FDA and MFDS), while it contained 49.8% (FDA) and 74.8% (MFDS) of iron.

#### 3.5.2. Amino-Acid Composition

The amino-acid composition of the puffed-rice snack enriched with drone pupae powder is summarized in Table 14, along with that of the control snack. The amino-acid contents of the developed product were higher than those of the control snack. In particular, the levels of isoleucine (0.19 g), lysine (0.16 g), valine (0.25 g), and glycine (0.22 g) for the developed product were approximately two times that of the control snack. These differences between the developed product and control were reasonable, as the drone pupae powder contained high levels of amino acids (Table 7). Kim et al. [44] have reported that bee drone pupae powder contains substantially high amounts of EAA, such as, isoleucine, lysine, and valine.

According to the same study [44], bee drone pupae powder contains high levels of glutamic acid; this is in agreement with the high value of glutamic acid recorded in the puffed-rice snack enriched with drone pupae powder in the present study. Comparing the results to daily intake requirements of WHO and the MOHW, the developed product showed higher percentages (7.7% and 11.6% in total) of EAA than the control product (4.2% and 6.3% in total).

#### 3.5.3. Fatty-Acid Composition

Table 15 summarizes the fatty-acid compositions (%) of the control and drone-pupae-powder enriched puffed-rice snack. Overall, the developed product exhibited a higher percentage (68.57%) of UFA than the control (67.83%) due to the high UFA content of the powder. Furthermore, the percentage of stearic acid (among the SFAs) was slightly higher in the developed product (6.47%) compared to the powder before processing (2.97%). According to a report of the RDA (2021) [48], drone pupae contains abundant phospholipids, from which stearic acid can be obtained by heating. In this study, the powder was mixed with the other ingredients (syrup and puffed rice) in the heated pot immediately after heating; thus, the mixing step could involve the aforementioned phenomenon.

Considering the UFA group, in the developed snack compared to the control, the content of oleic acid and linolenic acid increased from 31.1% to 35.01% and 1.39% to 3.05%, respectively, whereas the content of linoleic acid decreased from 34.29% to 29.69%. The drone pupae powder used for developing the final product was prepared using the deep-frying method with cooking oil; according to a study by Choi and Gil [49], deep-fried foods exhibit a long heating time and high oleic acid and low linoleic acid contents, regardless of the oil type used.

According to Ney [50], the rise of cholesterol levels by fatty acids is largely due to saturated fatty acids with 12, 14, and 16 carbons. In this study, these fatty acids did not increase, while there were high percentages of C18 fatty acids in the developed product. Overall, the puffed-rice snack enriched with drone pupae powder possessed a higher ratio of n-3 to n-6 fatty acids (n-3/n-6 value of 10.8%) than the control (4.5%). Hernandez and Hosokawa [51] have reported that, based on WHO recommendations, the n-3/n-6 value should be 1:5–1:10; thus, the product developed in this study exhibited a high nutritional value. 

#### 3.5.4. Minerals and Heavy Metals

Table 16 summarizes the mineral and heavy-metal contents of the control and drone-pupae-powder enriched puffed-rice snack. The results are shown as percentages of the international (Canada, USA, Europe) and MFDS (Korea) recommendations. The developed product containing drone pupae powder exhibited higher values (0.08, 0.02, and 0.35 mg) of phosphorus, magnesium, and copper compared to the control (a normal puffed-rice snack) (0.04, 0.01, and 0.23 mg, respectively), while the amount of zinc was lower in the developed product (1.62 mg) compared to the control (1.92 mg). Here, the drone-pupae-powder enriched product exhibited a very low amount of zinc (Table 9); this was investigated further.

Considering recommended amounts, magnesium and copper occurred under 10%, whereas phosphorus and zinc occurred in the range of 10–20%, regardless of sample group and recommendation source. The heavy metals exhibited the following values and percentages according to recommendations (MFDS; international): lead (0.01 mg; 10%; 10%), cadmium (0.01 mg; 10%; 20%), mercury (<0.01 mg; -; <2%), and arsenic (0.01 mg; 10%; 10%). Thus, the developed product is a safe and edible insect-based food.

### 3.6. Effects of Different Storage Conditions on the Quality of the Puffed-Rice Snack Enriched with Drone Pupae Powder

**Figure 6:**
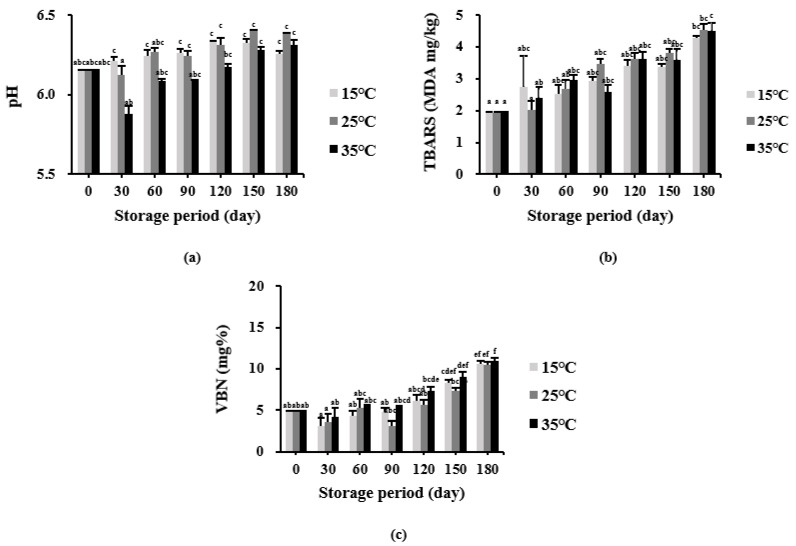
Chemical quality parameters of the puffed-rice snack enriched with drone pupae powder at different storage temperatures (15 °C, 25 °C, and 35 °C) for 180 days: (**a**) pH values; (**b**) TBARS values; (**c**) VBN values. Values are mean ± SD. Different letters (a–f) in the respective columns indicate significant differences among the means by the Tukey’s test (*p* < 0.05).

Figure 6 shows the changes in pH, TBARS, and VBN of the developed product for 180 days under different storage temperatures (15 °C, 25 °C, and 35 °C). As shown in Figure 6a, the storage conditions did not significantly change the pH values (6.26, 6.38, and 6.32 at 15 °C, 25 °C, and 35 °C, respectively) compared to the initial value (6.15), they were just slightly increased. All of the pH values were slightly acidic (5.0–6.5), similar to a previous study [52] reporting a rice-cracker product stored at different temperatures.

The TBARS values are shown in Figure 6b; it was 1.924 MDA mg/kg before storage, and significantly increased after 180 days at all storage temperatures. The final values were 4.287, 4.533, and 4.513 MDA mg/kg at 15 °C, 25 °C, and 35 °C, respectively. Thus, storage at 15 °C inhibit lipid oxidation to a greater extent than that at higher temperatures (such as, 25 °C and 35 °C). According to a study by Son et al. [53], insects with high fat content (such as bee drone pupae) show slightly increased TBARS values (lower than twice the initial value) after storage at fresh temperatures.

Figure 6c shows the VBN values for the storage period. After 180 days, the developed rice snack (using drone pupae powder) exhibited significantly higher VBN values at all temperatures (10.62 mg% at 15 °C, 10.50 mg% at 25 °C, and 10.97 mg% at 35 °C) compared to the initial value (4.8 mg%) (*p* < 0.05), and the values were all lower than 20 mg%, indicating acceptable VBN quality according to the MFDS. The drone pupae powder added to the developed product caused the change in VBN values during the storage period, similar to the increments in protein and amino-acid content (Table 13 and Table 14) discussed previously.

To assess microbial quality, the levels of TBC, *E. coli,* and TCG of the final product during storage have been summarized in Table 17. The TBC values did not exhibit a significant change (*p* < 0.05), whereas *E. coli* and TCG were not detected. The TBC values were below the recommended limit (5 log CFU/g) of the MFDS. This could be attributed to the low moisture content and sealed condition of the package, with low water activation conditions due to low relative humidity.

**Figure 7:**
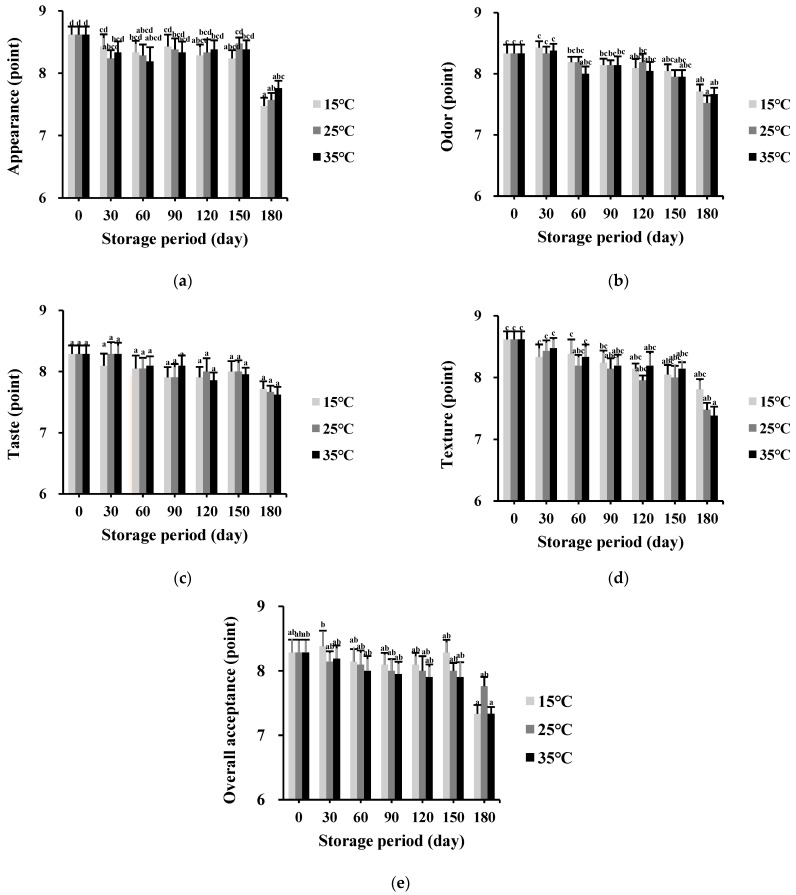
Changes in sensory evaluation of the puffed-rice snack enriched with drone pupae powder at different storage temperatures (15 °C, 25 °C, and 35 °C) for 180 days: (**a**) Appearance; (**b**) Odor; (**c**) Taste; (**d**) Texture; (**e**) Overall acceptance. The values are mean ± SD. Different letters (a–c) in the columns indicate significant differences among the means by the Tukey’s test (*p* < 0.05).

Figure 7 shows the sensory evaluation results of the puffed-rice snack (enriched with drone pupae powder) stored at different temperatures for 180 days by the hedonic scale method. The developed product maintained scores above 7-points during the storage period for all the sensory items, regardless of the storage temperature. Figure 7a shows the scores on appearance that changed from 8.48 (0 day) to 7.33, 7.76, and 7.33 at 15 °C, 25 °C, and 35 °C (180 days), respectively, with significant differences (*p* < 0.05). Interestingly, the appearance scores at each temperature rapidly decreased after 150 days. According to the sensory panelists, the final samples looked a bit yellowish and non-fresh after 180 days. The odor scores are shown in Figure 7b; similar to the appearance results, the tested products showed significantly lower points for odor than the initial state after 180 days under all storage conditions. As shown in Figure 6b, the samples stored for 180 days showed TBARS values above 4 MDA mg/kg; thus, the sensory qualities could be affected by lipid oxidation. However, the taste, texture, and overall acceptance scores did not change significantly after storage for 180 days at different temperatures, as shown in Figure 7c–e.

## 4. Conclusions

In this study, the best processing method for honeybee drone pupae powder and the development of a puffed-rice snack product using the powder has been described. The combination of deep-frying and hot-air drying was confirmed to be the best method for processing drone pupae to powder, considering various quality characteristics. Subsequently, a puffed-rice snack product enriched with the optimal powder (DHD) was prepared and its nutritional qualities and storage effect under different conditions were tested. The drone pupae powder exhibiting the best acid value and TBARS was selected among the different processing groups; it showed good sensory qualities (high nutty aroma and low bitter taste) and nutritional values considering institutional recommendations (high EAA and UFA). The drone pupae powder (5.54%) and honey (2.13%) were optimized using response surface methodology by adjusting the mixing concentrations with the puffed-rice snack to obtain the targeted nutty aroma and sweetness taste. The developed puffed-rice snack enriched with drone pupae powder exhibited higher values of nutrients (including proteins, fats, amino acids, and fatty acids) compared to the control rice snack. Additionally, the mineral content of the selected drone pupae powder and snack product were examined; heavy metals were not detected. In the storage test, the final product exhibited safe quality values considering important parameters (such as VBN and bacterial levels), along with a stable sensory likeness for 180 days. Therefore, the processed drone pupae powder is suitable to be used as an edible food ingredient. Furthermore, both the powder and the powder-enriched rice snack product exhibit high nutritional value and are safe for consumption. 

## Figures and Tables

**Table 1:** Factors and coded levels for the central composite design of processing the puffed-rice snack.

Independent Variables	Symbol	Unit	Range Level				
			−1.414	−1	0	+1	+1.414
Drone pupae powder	X_1_	%	2.172	3.0	5.0	7.0	7.828
Honey	X_2_	%	0.586	1.0	2.0	3.0	4.414

**Table 2:** The glossary of standards and their scores, defining the quality characteristics used in the sensory analysis.

Sensory Quality	Descriptive Terms	Definition	Standard and Scores
Appearance	Darkness color	Low illumination and absorbs light, such as black and brown.	*White sugar = 0, Grey oyster mushroom = 4, Black sesame = 10*
	Brownness color	Orange of low brightness and saturation.	*Curry powder = 3, Chocolate = 6, Americano coffee = 10*
Aroma	Nutty aroma	Containing, smelling of, or similar to nuts.	*Water = 0, Cheese powder = 6, Parched cereal powder = 8, Sesame oil = 10*
Taste	Sweetness taste ^1^	Perceived when eating foods rich in sugars	*Whipped cream = 5, Chocolate = 8, Honey = 10*
	Burnt taste	Overwhelmingly bitter and unpleasantly overshadowed by acridness.	*Water = 0, Grilled meat surface = 5, Over extracted-espresso = 10*
	Bitter taste	Sharp, pungent, or disagreeable flavor.	*Lettuce = 3, Grapefruit = 7, Ginseng = 10*
Texture	Sticky texture	Tending to hold like glue.	*Tofu (okara) powder = 5, Ketchup = 7, Caramel = 10*
	Rough texture	Uneven surface and not smooth.	*Flour = 1*, *White sugar = 6, Bread crumbs = 10*

^1^ Sweetness taste was used in the optimization of drone pupae powder and honey on processing the puffed-rice snack. The standards written in Italic font were used for the targeted scores in RSM.

**Table 3:** Sensory evaluation results by the descriptive test on drone pupae powders processed using different methods.

Descriptive Terms	UHD ^1^	SHD ^2^	DHD ^3^	UFD ^4^	SFD ^5^	DFD ^6^
Darkness color	6.25 ± 0.46 ^b^	5.75 ± 0.46 ^b^	7.38 ± 0.74 ^a^	2.75 ± 0.46 ^c^	3.00 ± 0.76 ^c^	8.00 ± 0.76 ^a^
Brownness color	5.63 ± 0.74 ^b^	3.50 ± 0.53 ^c^	8.88 ± 0.64 ^a^	1.50 ± 0.74 ^d^	2.38 ± 0.52 ^d^	8.13 ± 0.99 ^a^
Nutty aroma	6.13 ± 0.64 ^b^	6.25 ± 0.46 ^b^	7.38 ± 0.52 ^a^	4.75 ± 0.89 ^c^	5.63 ± 0.52 ^bc^	8.00 ± 0.76 ^a^
Burnt taste	2.63 ± 0.92 ^b^	3.88 ± 0.35 ^a^	4.50 ± 0.76 ^a^	2.13 ± 0.35 ^b^	2.38 ± 0.52 ^b^	4.50 ± 0.76 ^a^
Bitter taste	2.13 ± 0.64 ^b^	2.00 ± 0.93 ^b^	2.63 ± 0.92 ^b^	4.38 ± 0.74 ^a^	4.25 ± 0.71 ^a^	2.63 ± 0.52 ^b^
Sticky texture	2.63 ± 0.52 ^c^	2.75 ± 0.71 ^c^	6.25 ± 0.89 ^a^	1.25 ± 0.46 ^d^	1.13 ± 0.35 ^d^	5.13 ± 0.64 ^b^
Rough texture	2.50 ± 0.53 ^b^	2.63 ± 0.92 ^b^	5.25 ± 0.71 ^a^	2.75 ± 1.04 ^b^	3.63 ± 0.52 ^b^	6.00 ± 0.76 ^a^

Values are mean ± standard deviation. Different letters (a–d) in each column indicate significant differences among means by the Tukey’s test (*p* < 0.05). ^1^ Unheated and hot air-dried drone pupae powder. ^2^ Stir-fried and hot air-dried drone pupae powder. ^3^ Deep fried and hot air-dried drone pupae powder. ^4^ Unheated and freeze-dried drone pupae powder. ^5^ Stir-fried and freeze-dried drone pupae powder. ^6^ Deep fried and freeze-dried drone pupae powder.

**Table 4:** Yield results of the drone pupae powders processed using different methods.

Powders	Control Drone Pupae (Frozen)	UHD	SHD	DHD	UFD	SFD	DFD
Weight (g)	700	156 ± 2.65 ^d^	150 ± 3.61 ^d^	206 ± 2.65 ^b^	172 ± 2.00 ^c^	167 ± 1.00 ^c^	222 ± 2.65 ^a^
Yield (*w*/*w* %)	-	22.29 ± 0.38 ^d^	21.43 ± 0.52 ^d^	29.43 ± 0.38 ^b^	24.57 ± 0.29 ^c^	23.86 ± 0.14 ^c^	31.71 ± 0.38 ^a^

Values are mean ± SD. Different letters (a–d) in each row indicate significant differences among the means by the Tukey’s test (*p* < 0.05). Dash (-) indicates not performed.

**Table 5:** The color and odor intensity of drone pupae powders processed using different treatment methods.

Powders	Color				Odor Intensity (VCI)
	L	a	b	Δ*E*	
Control drone pupae	56.84 ± 0.67 ^a^	3.31 ± 0.11 ^e^	25.55 ± 0.45 ^a^	32.92 ± 0.73 ^a^	349.67 ± 2.5 ^d^
UHD	27.36 ± 0.22 ^d^	8.83 ± 0.08 ^a^	19.37 ± 0.10 ^b^	17.76 ± 0.12 ^c^	382.33 ± 1.53 ^b^
SHD	26.42 ± 0.09 ^e^	7.16 ± 0.02 ^c^	17.63 ± 0.04 ^c^	16.33 ± 0.04 ^e^	365.67 ± 1.53 ^c^
DHD	15.09 ± 0.05 ^g^	8.21 ± 0.06 ^b^	12.03 ± 0.06 ^e^	12.91 ± 0.03 ^g^	376.00 ± 2.00 ^bc^
UFD	41.28 ± 0.03 ^b^	2.26 ± 0.01 ^g^	12.02 ± 0.01 ^e^	21.87 ± 0.02 ^b^	453.67 ± 3.06 ^a^
SFD	33.62 ± 0.02 ^c^	2.96 ± 0.02 ^f^	12.88 ± 0.02 ^d^	16.90 ± 0.01 ^d^	336.00 ± 8.72 ^e^
DFD	21.07 ± 0.07 ^f^	5.82 ± 0.04 ^d^	11.41 ± 0.04 ^f^	13.19 ± 0.14 ^f^	302.67 ± 0.58 ^f^

Values are mean ± SD. Different letters (a–g) in each column indicate significant differences among the means by the Tukey’s test (*p* < 0.05). VCI indicates the volatile component intensity.

**Table 6:** The microbial qualities and moisture content of the control drone pupae and optimal drone pupae powder.

Samples	Total Bacteria Count (Log CFU/g)	*E. coli* and (CFU/g)	Total Coliform Group (CFU/g)	Moisture Content (*w*/*w* % Wet Basis)
Control drone pupae	2.56 ± 0.29 ^a^	-	-	51.9 ± 0.10 ^a^
Drone pupae powder (DHD)	2.30 ± 0.01 ^a^	-	-	4.54 ± 0.02 ^b^

Values are mean ± SD. Different letters (a,b) in each column indicate significant differences among the means by the *t*-test (*p* < 0.05). Dash (-) indicates not identified.

**Table 7:** The amino-acid composition of the optimal drone pupae powder.

Amino Acids	The Experimental Sample (g/100 g)			Recommended Daily Requirement	
	Amount	% WHO	% MOHW	WHO ^1^ (g/70 kg Body Weight)	MOHW ^2^ (g of 19–29 Ages/Male)
Histidine	0.92	87.6	92.0	1.05	1.0
Isoleucine	1.86	88.6	143.1	2.10	1.3
Leucine	2.89	70.0	96.3	4.13	3.0
Lysine	2.19	69.5	73.0	3.15	3.0
SAA ^3^	1.04	67.5	80.0	1.54	1.3
AAA ^4^	3.08	115.8	110.0	2.66	2.8
Threonine	1.28	79.5	91.4	1.61	1.4
Tryptophan	0.25	6.0	83.3	4.20	0.3
Valine	2.14	78.4	164.6	2.73	1.3
∑ EAA ^5^	15.65	67.5	101.6	23.17	15.4
Aspartate	3.40				
Serine	1.18				
Glutamic	5.43				
Proline	2.20				
Glycine	2.06				
Alanine	2.08				
Arginine	1.72				
∑ NAA ^6^	18.07				

^1^ According to the Protein and Amino Acid Requirements in Human Nutrition (WHO, 2007). ^2^ According to the Dietary Reference Intakes for Koreans (Ministry of Health and Welfare, MOHW; 2020). ^3^ SAA: sulfur amino acids (cysteine, methionine). ^4^ AAA: aromatic amino acids (tyrosine, phenylalanine). ^5^ EAA: essential amino acids. ^6^ NAA: nonessential amino acids.

**Table 8:** Fatty-acid compositions of the optimal drone pupae powder and other references for bee powder (% of total fatty acids).

Fatty Acids	The Experimental Sample	Ghosh et al. [41]	Kim et al. [39]
	Deep Fried and Hot Air-Dried	Oven-Dried	Freeze-Dried (−70~85 °C, 72 h)
	20 Days Old	21 Days within	16–20 Days Old
Caprylic acid	0.02	-	-
Capric acid	0.01	-	-
Lauric acid	0.09	0.4	0.64
Myristic acid	1.21	2.9	4.64
Pentadecanoic acid	0.03	-	-
Palmitic acid	24.22	35.1	35.49
Magaric acid	0.05	-	-
Stearic acid	6.79	12.6	14.46
Arachidic acid	0.21	-	0.74
Heneicosylic acid	-	-	-
Behenic acid	-	-	0.22
Tricosanoic acid	-	-	2.17
Lignoceric acid	0.02	-	1.26
∑ SFA	32.65	51.1	59.62
Myristoleic acid	0.02	-	-
Pentadecenoic acid	-	-	-
Palmitoleic acid	0.35	0.6	1.13
Magaoleic acid	0.05		-
Oleic acid	32.19	47.6	35.91
Eicosenoic acid	0.09	0.8	0.17
Eicosadienoic acid	0.01	-	-
Erucic acid	0.01	-	-
∑ MUFA	32.72	48.9	37.20
Linoleic acid	31.14	-	1.14
γ-Linolenic acid	-	-	-
Dihomo γ-Linolenic acid	0.02	-	-
Arachidonic acid	0.04	-	-
Docosadienoic acid	-	-	-
∑ n-6	31.2	-	1.14
Linolenic acid	3.34	-	2.04
Eicosatrienoic acid	-	-	-
Eicosapentaenoic acid	0.08	-	-
Docosapentaenoic acid	-	-	-
Docosahexaenoic acid	-	-	-
∑ n-3	3.42	-	2.04
∑ PUFA	34.62	-	3.18
∑ UFA	67.34	48.9	40.38
Total fatty acid (%)	100.0	100.0	100.0

Dash (-) means not measured/suggested.

**Table 9:** Analysis of the mineral and heavy-metal composition of the optimal drone pupae powder.

Minerals and Heavy Metals	Unit	Result	Amount % and Recommendations				Remark
			%	MFDS	%	International	
Sodium	mg/100 g	41.90	2.1	<2000 ^1^	1.8	<2300 ^2^	Following the standard of nutrition facts
Calcium	g/100 g	0.03	4.3	>0.7 ^1^	2.3	>1.3 ^2^	
Potassium	g/100 g	1.04	29.4	>3.5 ^1^	22.1	>4.7 ^2^	
Iron	mg/100 g	5	41.67	>12 ^1^	62.5	>8 ^2^	
Phosphorus	g/100 g	0.57	81.4	>0.7 ^3^	81.4	>0.7 ^4^	Following the standard of minerals
Magnesium	g/100 g	0.07	19.4	>0.36 ^3^	16.7	>0.42 ^4^	
Zinc	mg/100 g	4.44	44.4	>10 ^3^	40.4	>11 ^4^	
Copper	mg/100 g	1.19	12.5	<9.5 ^3^	11.9	<10 ^4^	
Lead	mg/kg	0.01	10	<0.1 ^5^	10	<0.1 ^6^	Following the standard of heavy metals
Cadmium	mg/kg	0.01	10	<0.1 ^5^	20	<0.05 ^7^	
Mercury	mg/kg	<0.01	-	-	<2	<0.5 ^8^	
Arsenic	mg/kg	0.01	10	<0.1 ^5^	10	<0.1 ^6^	

^1^ According to Nutrition Facts Labeling Requirements, US Food and Drug Administration. Industry Resources on the Changes to the Nutrition Facts Label. ^2^ According to Article 6 (Nutrition Facts Label) in Act on Labeling and Advertising of Foods (MFDS, 2020). ^3^ According to the Dietary Reference Intakes for Koreans (Ministry of Health and Welfare, MOHW; 2020). ^4^ According to the Dietary Reference Intakes (FDA, 2020). ^5^ According to the Food material recognition of MFDS (No. 2020-5). ^6^ According to the recommendation for metal amount in food (FDA, 2020). ^7^ According to the maximum limit for cadmium (European Community, 2005). ^8^ According to the maximum limit for mercury (Health Canada, 2019).

**Table 10:** Experimental results for central composite design.

Run No.	Coded Values		Actual Values		Responses	
	X_1_	X_2_	Drone Pupae Powder (X_1_)	Honey (X_2_)	Nutty Aroma (Y_1_)	Sweetness Taste (Y_2_)
1	−1	−1	3	1	8.00	7.88
2	+1	−1	7	1	9.13	7.13
3	−1	+1	3	3	6.13	8.50
4	+1	+1	7	3	7.13	8.25
5	−1.414	0	2.17	2	7.00	8.38
6	+1.414	0	7.83	2	8.25	7.38
7	0	−1.414	5	0.59	8.00	6.50
8	0	+1.414	5	4.41	7.13	8.50
9	0	0	5	2	8.13	8.00
10	0	0	5	2	7.88	8.13
11	0	0	5	2	7.88	7.88

**Table 11:** Analysis of variance for responses on the optimization of mixing conditions for drone pupae powder and honey with a puffed-rice snack: X_1_, drone pupae powder; X_2_, honey; Y_1_, nutty aroma; Y_2_, sweetness taste.

Responses	R^2^	Lack of Fit (>0.05)	*p*-Values (<0.05)			
			Model	Linear (X_1_, X_2_)	Quadratic (X_1_X_1_, X_2_X_2_)	Interaction (X_1_X_2_)
Y_1_ (nutty aroma)	0.854	0.087	>0.011	0.004, 0.013	>0.526, 0.821	-
Y_2_ (sweetness taste)	0.902	0.155	>0.001	0.015, 0.001	>0.970, 0.165	-

Dash (-) means not identified.

**Table 12:** Optimization of the mixing conditions for drone pupae powder and honey with a puffed-rice snack by RSM.

Responses	Optimal Conditions		Predicted Values	Experimental Values	Desirability
	X_1_ (%)	X_2_ (%)			
Y_1_ (nutty aroma)	+0.271 (5.54%)	+0.129 (2.13%)	7.992	8.19 ± 0.40	0.9714
Y_2_ (sweetness taste)			7.997	8.43 ± 0.51	0.9183

**Table 13:** Fourteen nutrients in the control and drone-pupae-powder enriched puffed-rice snack.

Items	The puffed Rice Snack						Daily Values	
	Control			Enriched with Drone Pupae Powder				
	Amount	% FDA	% MFDS	Amount	% FDA	% MFDS	FDA ^1^	MFDS ^2^
Calories (cal)	375.14	-	-	389.9	-	-	-	-
Sodium (mg)	15.22	0.7	0.8	12.87	0.6	0.6	<2300	<2000
Carbohydrate (g)	89.04	32.4	27.5	82.65	30.1	25.5	>275	>324
Sugar (g)	44.28	88.6	44.3	38.18	76.4	38.2	<50	<100
Dietary fiber (g)	1.33	4.8	5.3	1.44	5.1	5.8	>28	>25
Crude fat (g)	1.06	1.4	2.0	4.60	5.9	8.5	<78	<54
Trans fat (g)	-	-	-	-	-	-	<2	-
Saturated fat (g)	0.34	1.7	2.3	1.44	7.2	9.6	<20	<15
Cholesterol (mg)	-	-	-	17.16	5.7	5.7	<300	<300
Crude protein (g)	2.36	4.7	4.3	4.45	8.9	8.1	>50	>55
Vitamin D (μg)	-	-	-	-	-	-	>20	>10
Potassium (g)	0.04	0.9	1.1	0.10	2.1	2.9	>4.7	>3.5
Iron (mg)	8.54	47.4	71.2	8.97	49.8	74.8	>18	>12
Calcium (g)	0.02	1.5	2.9	0.01	0.8	1.4	>1.3	>0.7

^1^ According to the Nutrition Facts Labeling Requirements, US Food and Drug Administration. ^2^ According to Article 6 (Nutrition Facts Label) in the Act on Labeling and Advertising of Foods (MFDS, 2020). Dash (-) means not identified.

**Table 14:** The total amino-acid composition of the control and drone-pupae-powder enriched puffed-rice snack.

Amino Acids	The Puffed Rice Snack (g/100 g)					
	Control			Enriched with Drone Pupae Powder		
	Amount	% WHO	% MOHW	Amount	% WHO	% MOHW
Histidine	0.06	5.7	6.0	0.10	9.5	10.0
Isoleucine	0.08	3.8	6.2	0.19	9.0	14.6
Leucine	0.20	4.8	6.7	0.35	8.5	11.7
Lysine	0.07	2.2	2.3	0.16	5.1	5.3
SAA	0.11	7.1	8.5	0.15	9.7	11.5
AAA	0.22	8.3	7.9	0.39	14.7	13.9
Threonine	0.09	5.6	6.4	0.16	9.9	11.4
Tryptophan	0.02	0.5	6.7	0.03	0.7	10.0
Valine	0.12	4.4	9.2	0.25	9.2	19.2
∑ EAA	0.97	4.2	6.3	1.78	7.7	11.6
Aspartate	0.22			0.40		
Serine	0.14			0.21		
Glutamic	0.47			0.75		
Proline	0.14			0.25		
Glycine	0.11			0.22		
Alanine	0.15			0.26		
Arginine	0.19			0.28		
∑ NAA	1.42			2.37		

**Table 15:** Fatty-acid compositions of the control and drone-pupae-powder enriched puffed-rice snack (% of total fatty acids).

Fatty Acids	Shorthand	The Puffed Rice Snack	
		% Control	% Enriched with Drone Pupae Powder
Caprylic acid	C8:0	0.15	0.03
Capric acid	C10:0	0.08	0.01
Lauric acid	C12:0	0.25	0.10
Myristic acid	C14:0	1.14	1.15
Pentadecanoic acid	C15:0	0.18	0.02
Palmitic acid	C16:0	26.92	23.24
Magaric acid	C17:0	0.17	0.09
Stearic acid	C18:0	2.97	6.47
Arachidic acid	C20:0	0.22	0.28
Heneicosylic acid	C21:0	-	-
Behenic acid	C22:0	-	-
Lignoceric acid	C24:0	0.07	0.04
∑ SFA		32.15	31.43
Myristoleic acid	C14:1	0.06	0.02
Pentadecenoic acid	C15:1	0.05	0.01
Palmitoleic acid	C16:1	0.43	0.35
Magaoleic acid	C17:1	0.09	0.03
Oleic acid	C18:1	31.10	35.01
Eicosenoic acid	C20:1	0.15	0.14
Eicosadienoic acid	C20:2	-	0.02
Erucic acid	C22:1	0.09	0.02
∑ MUFA		31.97	35.6
Linoleic acid	C18:2 n-6	34.29	29.69
γ-Linolenic acid	C18:3 n-6	0.03	0.01
Dihomo γ-Linolenic acid	C20:3 n-6	-	0.03
Arachidonic acid	C20:4 n-6	-	0.03
∑ n-6		34.32	29.76
Linolenic acid	C18:3 n-3	1.39	3.05
Eicosatrienoic acid	C20:3 n-3	-	-
Eicosapentaenoic acid (EPA)	C20:5 n-3	0.10	0.13
Docosapentaenoic acid (DPA)	C22:5 n-3	-	-
Docosahexaenoic acid (DHA)	C22:6 n-3	0.05	0.03
∑ n-3		1.54	3.21
n-3/n-6 (%)		4.5	10.8
∑ PUFA		35.86	32.97
∑ UFA		67.83	68.57
Total fatty acid (%)		100	100

**Table 16:** The mineral and heavy-metal composition of the control and drone-pupae-powder enriched puffed-rice snack.

Minerals and Heavy Metals	Unit	The Puffed Rice Snack					
		Control			Enriched with Drone Pupae Powder		
		Amount	% MFDS	% Int’l	Amount	% MFDS	% Int’l
Phosphorus	g/100 g	0.04	6.7	6.7	0.08	10.9	10.9
Magnesium	g/100 g	0.01	2.7	2.3	0.02	4.1	3.5
Zinc	mg/100 g	1.92	19.2	17.5	1.62	16.2	14.7
Copper	mg/100 g	0.23	2.4	2.3	0.35	3.7	3.5
Lead	mg/kg	0.01	10	10	0.01	10	10
Cadmium	mg/kg	0.01	10	20	0.01	10	20
Mercury	mg/kg	<0.01	-	<2	<0.01	-	<2
Arsenic	mg/kg	0.01	10	10	0.01	10	10

**Table 17:** Changes in the total bacteria count (TBC), *E. coli*, and total coliform group (TCG) in the puffed-rice snack enriched with drone pupae powder at different storage temperatures (15 °C, 25 °C, 35 °C) for 180 days.

Temperature	Day	Total Bacteria Count (Log CFU/g)	*E.coli* and Total Coliform Group (CFU/g)
15 °C	0	1.57 ± 0.09 ^a^	-
	30	1.29 ± 0.21 ^a^	-
	60	1.37 ± 0.15 ^a^	-
	90	1.22 ± 0.20 ^a^	-
	120	1.39 ± 0.07 ^a^	-
	150	1.25 ± 0.10 ^a^	-
	180	1.41 ± 0.19 ^a^	-
25 °C	0	1.57 ± 0.09 ^a^	-
	30	1.35 ± 0.13 ^a^	-
	60	1.22 ± 0.20 ^a^	-
	90	1.37 ± 0.05 ^a^	-
	120	1.29 ± 0.09 ^a^	-
	150	1.10 ± 0.14 ^a^	-
	180	1.28 ± 0.14 ^a^	-
35 °C	0	1.57 ± 0.09 ^a^	-
	30	1.53 ± 0.10 ^a^	-
	60	1.29 ± 0.09 ^a^	-
	90	1.16 ± 0.12 ^a^	-
	120	1.26 ± 0.20 ^a^	-
	150	1.29 ± 0.21 ^a^	-
	180	1.10 ± 0.14 ^a^	-

Values are mean ± SD. The small letter (a) in a column indicates the significant difference among means by the Tukey’s test (*p* < 0.05). Dash (-) indicates not detected.

## Data Availability

Data supporting the reported results are available upon request.

## References

[1] Choi J.S. (2021). Nutrition, Safety, Health Functional Effects, and Availability of Honeybee (*Apis mellifera* L.) Drone Pupae. Insects.

[2] Evans J., Müller A., Jensen A.B., Dahle B., Flore R., Eilenberg J., Frøst M.B. (2016). A descriptive sensory analysis of honeybee drone brood from Denmark and Norway. J. Insects Food Feed..

[3] Han S.M., Moon H.J., Woo S.O., Bang K.W., Kim S.G., Kim H.Y., Choi H.M., Lee M.Y. (2019). Bee Pupa Cookbook Nutritious, Bee Pupa Cooking.

[4] Kulma M., Tůmová V., Fialová A., Kouřimská L. (2020). Insect consumption in the Czech Republic: What the eye does not see, the heart does not grieve over. J. Insects Food Feed..

[5] Wilkinson K., Muhlhausler B., Motley C., Crump A., Bray H., Ankeny R. (2018). Australian consumers’ awareness and acceptance of insects as food. Insects.

[6] Winston M.L. (1991). The Biology of the Honey Bee.

[7] Ulmer M., Smetana S., Heinz V. (2020). Utilizing honeybee drone brood as a protein source for food products: Life cycle assessment of apiculture in Germany. Resour. Conserv. Recy..

[8] Krell R. (1996). Value-Added Products from Beekeeping.

[9] Ministry of Food and Drug Safety Status of Temporary Recognition of Standards and Specifications for Food Ingredients. 44. Honeybee Drone Pupae (*Apis mellifera* L.). https://www.foodsafetykorea.go.kr/portal/board/boardDetail.do.

[10] Ramírez-Rivera E.J., Hernández-Santos B., Juárez-Barrientos J.M., Torruco-Uco J.G., Ramírez-Figueroa E., Rodríguez-Miranda J. (2021). Effects of formulation and process conditions on chemical composition, color parameters, and acceptability of extruded insect-rich snack. J. Food Processing Preserv..

[11] García-Gutiérrez N., Mellado-Carretero J., Bengoa C., Salvador A., Sanz T., Wang J., Ferrando M., Güell C., Lamo-Castellví S.D. (2021). ATR-FTIR Spectroscopy Combined with Multivariate Analysis Successfully Discriminates Raw Doughs and Baked 3D-Printed Snacks Enriched with Edible Insect Powder. Foods.

[12] Roncolini A., Milanović V., Aquilanti L., Cardinali F., Garofalo C., Sabbatini R., Clementi F., Belleggia L., Pasquini M., Mozzon M. (2020). Lesser mealworm (*Alphitobius diaperinus*) powder as a novel baking ingredient for manufacturing high-protein, mineral-dense snacks. Food Res. Int..

[13] Severini C., Azzollini D., Albenzio M., Derossi A. (2018). On printability, quality and nutritional properties of 3D printed cereal based snacks enriched with edible insects. Food Res. Int..

[14] Gallagher E. (2009). Gluten-Free Food Science and Technology.

[15] We G.J., Lee I.A., Cho Y.S., Yoon M.R., Shin M.S., Ko S.H. (2010). Development of rice flour-based puffing snack for early childhood. Food Eng. Prog..

[16] Kang S.M., Maeng A.R., Seong P.N., Kim J.H., Cho S., Kim Y., Choi Y.S. (2018). Effect of drone pupa meal added as replacement of sodium nitrite and vitamin C on physico-chemical quality characteristics of emulsion-type sausage. Korean J. Food Nutr..

[17] Kang S.I., Kim K.H., Lee J.K., Kim Y.J., Park S.J., Kim M.W., Choi B.D., Kim D., Kim J.S. (2014). Comparison of the food quality of freshwater rainbow trout *Oncorhynchus mykiss* cultured in different regions. Korean J. Fish. Aquat. Sci..

[18] Ministry of Food and Drug Safety (MFDS) (2021). 8th General Analysis Method. Food Code (Sik-Poom-Gong-Jeon).

[19] Buege J.A., Aust S.D. (1978). MDA levels in plasma and TBARs. Methods Enzym..

[20] (2016). Sensory Analysis—Methodology—General Guidance for Establishing a Sensory Profile.

[21] AOAC International (2000). International Official Methods of Analysis Official Methods 994.12.

[22] Bligh E.G., Dyer W.J. (1959). The diet of amphioxus in subtropical Hong Kong as indicated by fatty acid and stable isotopic analyses. Can. J. Biochem. Physiol..

[23] Official Methods and Recommended Practices of the AOCS (2006). AOCS Official Method Ce 1c-89.

[24] American Oil Chemists’ Society (2006). AOCS Official Method Cd 14c-94.

[25] AOAC International (2000). International Official Methods of Analysis Official Methods 925.09, 923.03, 979.09, 962.09, and 923.05.

[26] AOAC International (2002). Method of Analysis–Official methods 990.12. International Official Methods of Analysis Official Methods.

[27] AOAC International (2002). Method of Analysis–Official methods 991.14. International Official Methods of Analysis Official Methods.

[28] Lin C.Y., Lin Y.C., Kuo H.K., Hwang J.J., Lin J.L., Chen P.C., Lin L.Y. (2009). Association among acrylamide, blood insulin, and insulin resistance in adults. Diabetes Care.

[29] Suderman D.R., WIKER J., Cunningham F.E. (1981). Factors affecting adhesion of coating to poultry skin: Effects of various protein and gum sources in the coating composition. J. Food Sci..

[30] Hwang S.Y., Kim G.S. (2020). A Study on Quality Characteristics of Silkworm (*Bombyx mori* L.) by various pretreatment methods. Culin. Sci. Hosp. Res..

[31] Pyo S.J., Jung C.E., Sohn H.Y. (2020). Platelet aggregatory and antidiabetic activities of larvae, pupae, and adult of honeybee drone (*Apis mellifera*). J. Apic..

[32] Andrikopoulos N.K., Dedoussis G.V., Falirea A., Kalogeropoulos N., Hatzinikola H.S. (2002). Deterioration of natural antioxidant species of vegetable edible oils during the domestic deep-frying and pan-frying of potatoes. Int. J. Food Sci. Nutr..

[33] Guillen-Sans R., Guzman-Chozas M. (1998). The thiobarbituric acid (TBA) reaction in foods: A review. Crit. Rev. Food Sci. Nutr..

[34] Koh H.Y., Kowon Y.J. (1989). Effect of storage temperature and humidity on water absorption and rancidity of peanuts. J. Korean Soc. Food Nutr..

[35] AL-KAHTANI H.A., ABU-TARBOUSH H.M., BAJABER A.S., ATIA M., ABOU-ARAB A.A., EL-MOJADDIDI M.A. (1996). Chemical changes after irradiation and post-irradiation storage in tilapia and Spanish mackerel. J. Food Sci..

[36] Santos M.S. (1996). Biogenic amines: Their importance in foods. Int. J. Food Microbiol..

[37] Jin S.K., Kim I.S., Hah K.H., Hur S.J., Lyou H.J., Park K.H., Bae D.S. (2005). Changes of qualities in aerobic packed ripening pork using a Korea traditional seasoning during storage. J. Anim. Sci. Technol..

[38] Kim J.E., Kim S.G., Kang S.J., Kim Y.H. (2016). Physicochemical Properties of the Bee Pupa and Main Pollen, Symposium for the Development of the Beekeeping Industry, Korea, 20–21 October 2016.

[39] Kim J.E., Kim D.I., Koo H.Y., Kim H.J., Kim S.Y., Lee Y.B., Kim J.S., Kim H.H., Moon J.H., Choi Y.S. (2020). Analysis of Nutritional Compounds and Antioxidant Effect of Freeze-Dried powder of the Honey Bee (*Apis mellifera* L.) Drone (Pupal stage). Korean J. Appl. Entomol..

[40] Ghosh S., Sohn H.Y., Pyo S.J., Jensen A.B., Meyer-Rochow V.B., Jung C. (2020). Nutritional composition of *Apis mellifera* drones from Korea and Denmark as a potential sustainable alternative food source: Comparison between developmental stages. Foods.

[41] Ghosh S., Jung C., Meyer-Rochow V.B. (2016). Nutritional value and chemical composition of larvae, pupae, and adults of worker honey bee, *Apis mellifera ligustica* as a sustainable food source. J. Asia-Pac. Entomol..

[42] Lee J.S., Cho S.Y., Choi S.G. (1996). Application of Soybean Oil in the Food Industry, Food Industry and Nutrition, Korea, December 1996, Busan.

[43] Recommended Intakes of Macronutrients (Nordic Nutrition Recommendations 2012). http://norden.diva-portal.org/smash/get/diva2:704251/FULLTEXT01.pdf.

[44] Kim S.G., Woo S.O., Bang K.W., Jang H.R., Han S.M. (2018). Chemical composition of drone pupa of *Apis mellifera* and its nutritional evaluation. Korean J. Apic..

[45] Aparna A.R., Rajalakshmi D. (1999). Honey—its characteristics, sensory aspects, and applications. Food Rev. Int..

[46] Jensen A.B., Evans J., Jonas-Levi A., Benjamin O., Martinez I., Dahle B., Roos N., Lecocq A., Foley K. (2019). Standard methods for *Apis mellifera* brood as human food. J. Apic. Res..

[47] Biró B., Sipos M.A., Kovács A., Badak-Kerti K., Pásztor-Huszár K., Gere A. (2020). Cricket-Enriched Oat Biscuit: Technological Analysis and Sensory Evaluation. Foods.

[48] Monthly Agricultural Technology (Nongsaro Agricultural Technology Portal in Rural Development Administration). http://www.nongsaro.go.kr/portal/ps/psv/psvr/psvre/curationDtl.ps?menuId=PS03352&srchCurationNo=1689&totalSearchYn=Y.

[49] Choi E.S., Gil B.I. (2011). Effects of Thermooxidation of Soybean Oil in Association with Fried Foods on Quantity Food Production. J. East Asian Soc. Diet. Life.

[50] Ney D.M. (1991). Potential for enhancing the nutritional properties of milk fat. J. Dairy Sci..

[51] Hernandez E., Hosokawa M. (2015). Omega-3 Oils: Applications in Functional Foods.

[52] Gunaratne T.M., Gunaratne N.M., Navaratne S.B. (2015). Selection of best packaging method to extend the shelf life of rice crackers. Int. J. Sci. Eng. Res..

[53] Son Y.J., Ahn W., Kim S.H., Park H.N., Choi S.Y., Lee D.G., Kim A.N., Hwang I.K. (2016). Study on the oxidative and microbial stabilities of four edible insects during cold storage after sacrificing with blanching methods. Korean J. Food Nutr..

